# Large-scale functional networks connect differently for processing words and symbol strings

**DOI:** 10.1371/journal.pone.0196773

**Published:** 2018-05-02

**Authors:** Mia Liljeström, Johanna Vartiainen, Jan Kujala, Riitta Salmelin

**Affiliations:** 1 Department of Neuroscience and Biomedical Engineering, Aalto University, Espoo, Finland; 2 Aalto NeuroImaging, Aalto University, Espoo, Finland; Eberhard-Karls-Universitat Tubingen Medizinische Fakultat, GERMANY

## Abstract

Reconfigurations of synchronized large-scale networks are thought to be central neural mechanisms that support cognition and behavior in the human brain. Magnetoencephalography (MEG) recordings together with recent advances in network analysis now allow for sub-second snapshots of such networks. In the present study, we compared frequency-resolved functional connectivity patterns underlying reading of single words and visual recognition of symbol strings. Word reading emphasized coherence in a left-lateralized network with nodes in classical perisylvian language regions, whereas symbol processing recruited a bilateral network, including connections between frontal and parietal regions previously associated with spatial attention and visual working memory. Our results illustrate the flexible nature of functional networks, whereby processing of different form categories, written words vs. symbol strings, leads to the formation of large-scale functional networks that operate at distinct oscillatory frequencies and incorporate task-relevant regions. These results suggest that category-specific processing should be viewed not so much as a local process but as a distributed neural process implemented in signature networks. For words, increased coherence was detected particularly in the alpha (8–13 Hz) and high gamma (60–90 Hz) frequency bands, whereas increased coherence for symbol strings was observed in the high beta (21–29 Hz) and low gamma (30–45 Hz) frequency range. These findings attest to the role of coherence in specific frequency bands as a general mechanism for integrating stimulus-dependent information across brain regions.

## Introduction

Large-scale functional networks are thought to arise through synchronization between distantly situated and functionally specialized brain regions [1, 2]. To support behavior, such networks should be flexibly reconfigured in a stimulus-specific and task-relevant manner [3, 4]. Here we focus on cortical connectivity supporting visual recognition of written words, as compared with processing of symbols, to assess formation of transient large-scale functional networks in response to different form categories.

Functional neuroimaging studies, using local activation measures, have identified multiple cortical regions in the left-hemisphere perisylvian language regions and in the occipito-temporal cortex that are involved at different stages of reading [5–8], but very few studies have investigated the intercommunication between these regions. In those studies that have focused on functional connectivity, the left occipito-temporal region has been identified as a central driving node within the reading network [9, 10]. Analysis of connectivity between pre-specified regions have further suggested that the left occipito-temporal, left superior temporal, and left inferior frontal or orbitofrontal cortex are interconnected during reading [9–11].

This reading network is thought to be formed through the rewiring of visual and language systems as reading skills are acquired [12]. Visual objects, such as symbols and written words, are represented along the ventral visual pathway in the ventral and lateral occipito-temporal cortex [13], in regions that respond preferentially to specific stimulus categories [14, 15]. Such regions within the ventral stream develop with expertise, even in the absence of visual experience [16], and it has been suggested that their locations can be predicted by their structural connectivity [17]. For reading, the region within the ventral temporal cortex (‘visual word form area’, or VWFA) that is important for visual recognition of letters and words develops as children (or adult illiterates) learn to read [18]. Several studies have focused on the structural connectivity of this region and shown that it has anatomical connections with the left-hemisphere perisylvian language regions [19, 20].

If it is the case that such category-specific regions emerge because of their inherent network connections [17], then we should also be able to show that these networks are indeed utilized in a stimulus-specific manner when those categories are being processed. Here, we chose two visual categories, words and symbols, and focused on modulations in connectivity within the large-scale networks that are involved in visual recognition of these two categories. Within the ventral pathway, activation studies have indicated that reading is more left-lateralized, whereas object-processing is bilateral. We thus expect that word reading should modulate left-hemisphere language circuits, while processing of symbols should involve a bilateral visual recognition network.

Many connectivity studies use pre-specified regions of interest in their analysis. Yet, language is a complex cognitive process that involves a large number of both language-specific and domain-general brain regions [21]. Therefore, we chose to characterize functional connectivity in an all-to-all connectivity approach without pre-specified regions. To this end, we combined time-sensitive magnetoencephalography (MEG) recordings with a spatial filtering technique that allowed us to focus on a sub-second time window (50–800 ms) following stimulus presentation [3, 22] and identify coherent large-scale networks at their characteristic frequency of operation. In connectivity analysis, regions that are part of a common network may show high coherence, even though these components of the network are not relevant to the particular task at hand. We aimed to identify those functional connections that were modulated by our specific stimulus conditions. To achieve this, we contrasted the coherence results for the two conditions.

## Materials and methods

### Subjects

Fifteen right-handed, native Finnish-speaking subjects (seven females; age 20–49 years, mean age 27 years) participated in the experiment. The participants all had normal, or corrected-to-normal vision and reported no neurological or language-related disorders. The participants signed a written informed consent form, in agreement with the prior approval of the Helsinki and Uusimaa Ethics Committee.

### Stimuli, measurement sessions, and experimental design

**Fig 1:**
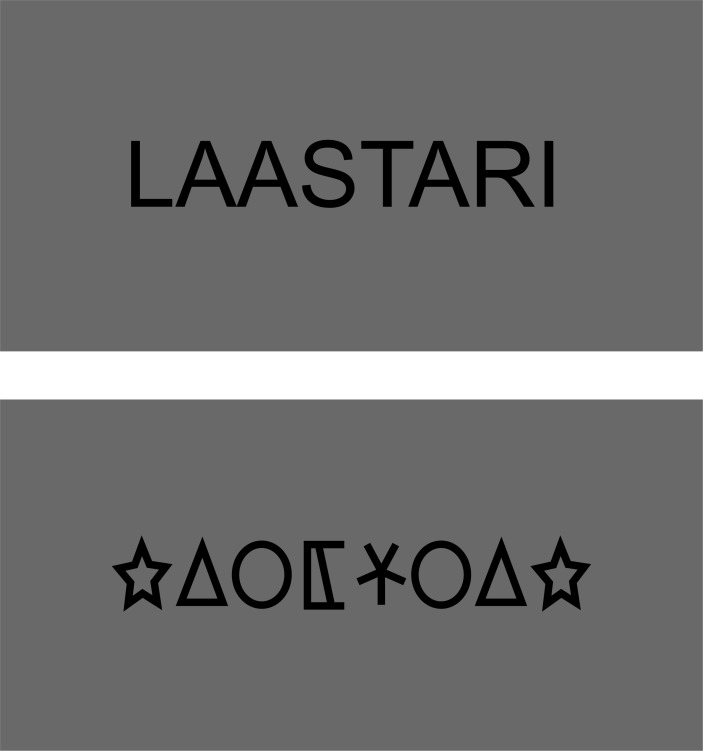
Example stimuli. The stimuli consisted of real Finnish nouns (above) and symbol strings (below). Stimulus categories were matched for visual complexity.

The stimuli were real Finnish nouns and symbol strings consisting of 10 different symbols (Fig 1). There were 112 unique stimuli within each category (length 7–8 letters or symbols), each presented once during the experiment. Symbols were selected randomly to form symbol strings where the same symbol appeared maximally twice in a row (Fig 1). The stimulus categories were matched for length and visual complexity. The stimuli were shown visually one at a time (visual angle < 7°). Each stimulus was presented for 300 ms, followed by 1200 ms of gray background. The stimuli were presented in a blocked design with 7 stimuli of the same category in each block. MEG data for stimulus blocks containing other visual categories were also recorded [23] but not included in the present analysis. The stimuli were shown in a random order within the blocks, and blocks of different stimulus categories were presented in a partially counterbalanced order. The first stimulus of each block was excluded from the analysis to avoid attention-related effects. To keep the participants alert and ensure that they were attending to the stimuli they were asked to indicate with a button press when the same stimulus appeared twice in a row. Target blocks (one per stimulus category, altogether five blocks during the entire experiment) were not included in the analysis.

### Data acquisition and preprocessing

MEG recordings were conducted in a magnetically shielded room using a Vectorview™ MEG device (Elekta, Helsinki, Finland), housing 204 gradiometer and 102 magnetometer channels. MEG signals were filtered at 0.03–200 Hz and sampled at 600 Hz. Vertical and horizontal electro-oculograms (EOG) were measured to identify eye blinks and saccades. Head position indicator coils were used to determine the position of the head with respect to the MEG sensors. The positions of the indicator coils with respect to anatomical landmarks (nasion and preauricular points) were identified with a 3D digitizer (Polhemus, Colchester, VT). Spatiotemporal signal space separation (tSSS) was applied to remove external disturbances and artefacts [24]. MEG epochs within the time window 50–800 ms after stimulus onset that contained eye blinks or saccades were rejected (EOG rejection limit 150 μV). After rejection, on average 77 trials remained in both conditions across subjects (no statistical difference between conditions; paired t-test, p = 0.92).

**Fig 2:**
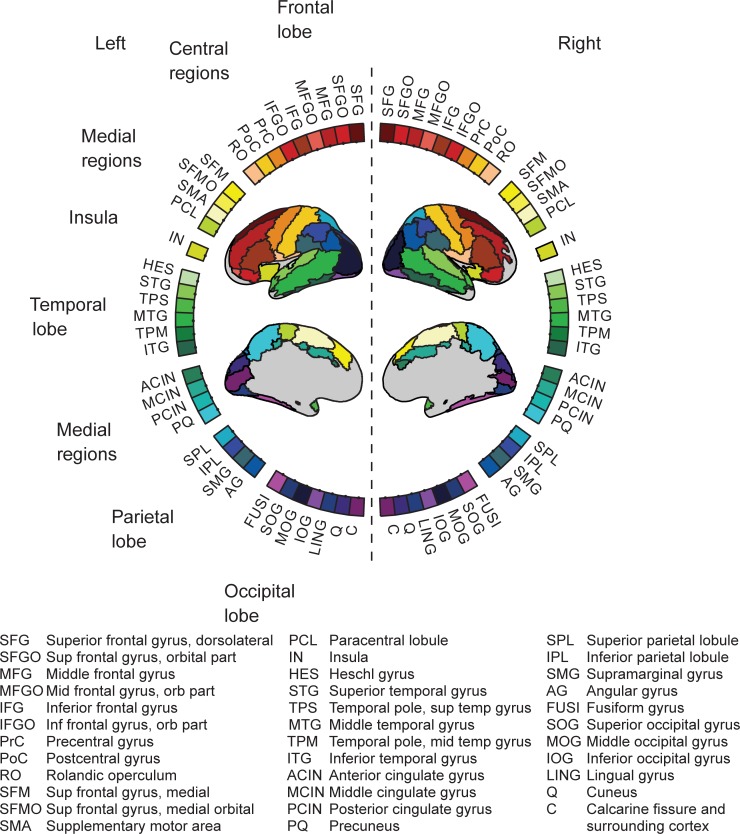
MEG connectogram. Network nodes obtained from an anatomical brain atlas (AAL; [31]) are illustrated on a circular representation of the cortex.

Structural MRIs were acquired with a 3 Tesla Signa EXCITE MRI scanner (GE Healthcare) using a T1-weighted 3D SPGR sequence with 0.9 × 0.9 × 1.0 mm^3^ resolution. For each participant, the cortical surface was reconstructed from the MRIs using the FreeSurfer software. For the purpose of the connectivity analysis, we created anatomical grids that were spatially equivalent across subjects. A surface-based grid with 9-mm spacing along the surface was created for one subject and then transformed using FreeSurfer to each subject's brain. Grid points that were located more than 7 cm from the closest sensor in this example subject were excluded from analysis. In addition, grid points in the temporal pole and in the anterior frontal cortex that are sensitive to eye movement artefacts in the MEG data were excluded. As a result, 3414 grid points of the original 5241 covering the entire gray matter surface were used in the analysis. Fig 2 depicts the excluded brain regions in light grey color.

### MEG connectivity analysis

We performed an all-to-all connectivity analysis to identify modulations in large-scale functional networks between experimental conditions [3]. Source regions with coherent time courses of activity were found using a spatial filter in the frequency domain (DICS, dynamic imaging of coherent sources [22, 25]). We used a wavelet-based version of the spatial filter [26] to obtain coherence estimates in a time interval of 50–800 ms after stimulus onset. A limiting factor in choosing the length of the time window was the need to obtain enough data points for a stable estimation of the data covariance matrix; this precluded the use of shorter time windows. Furthermore, the time window needs to be sufficiently long to allow evaluation of effects at lower frequencies (e.g., in the theta band). All analysis was performed with in-house matlab scripts that we have developed for spatial filtering and connectivity analysis of MEG data [3, 22, 25, 26].

Data were analyzed in six frequency bands of interest: 3–7 Hz (theta), 8–13 Hz (alpha), 15–20 Hz (low beta), 21–29 Hz (high beta), 30–45 Hz (low gamma), and 60–90 Hz (high gamma) (similarly to [3]). Within this approach, we first calculated a time-frequency representation for data epochs ranging from -250 to 900 ms with respect to stimulus onset using Morlet wavelets of width 7 at the sensor level in the frequency range 3–90 Hz, at 2-Hz intervals. This time-frequency representation was calculated by convolving the wavelets and the data epochs. Cross-spectral density matrices (CSDs) across all planar gradiometer channels were then calculated using this time-frequency representation, and averaged over the desired 50–800 ms time interval. Only gradiometers were used as they have a narrow spatial sensitivity pattern and are optimal for recording data from superficial sources; for studying such sources, magnetometers that more readily pick up signals from distant sources and external artefacts would have been less beneficial. In addition, the tSSS procedure renders source estimation from gradiometer and magnetometer data highly similar [27]. The CSD matrices were averaged over the frequency bands of interest. The sensor-level data, represented by the CSD matrix, were then transformed into a cortical representation using the spatial filter. Coherence estimation was based on numerical maximization of coherence for a discrete set of combinations of the source current orientations [3, 4]. For each connection, the configuration of the source current orientations was determined by identifying the orientation combination that maximized the mutual coherence of the two sources. The orientation pairs were tested for all possible combinations of 50 regularly spaced orientations (spanning 180°, in steps of 3.6°) in the tangential source space of a sphere that best approximated the curvature of the inner surface of the skull. The numerical maximization was done separately for the two conditions, with distinct CSD matrices. This approach enables the identification of condition-specific orientation combinations for each connection, facilitating sensitive determination of coherence also for sources that show weak activity levels. The source reconstruction utilized a spherical head conductor model, determined individually for each subject. The head model was fitted to the shape of the inner surface of the skull based on the subject’s MRI.

Power differences between conditions can introduce spurious differences in the coherence estimates [28]. The frequency bands were therefore tested at the sensor-level to exclude frequency bands with large power differences between stimulus categories (amplitude difference between conditions exceeding 10%, see [29]. We estimated the mean power within each frequency band of interest and tested for power differences separately at each sensor to ensure that the spatial power pattern was similar in both conditions [30]. Frequency bands with power differences at 5 or more sensors were excluded from further analysis.

In the accepted frequency bands, coherence estimates for epochs of 50–800 ms after the stimulus onset were computed for each anatomical grid point with all other grid points in the frequency bands of interest (all-to-all connectivity) [3, 4]. A minimum distance limit of 4 cm between the connection start and end points was applied to avoid spurious connectivity between closely situated areas [3, 28]. To establish stimulus-specific modulations in connectivity, we contrasted the connectivity results for words and symbol strings (p<0.05, FWE corrected, see below for statistical evaluation). Our results therefore describe modulations of connectivity between stimulus categories, rather than the entire underlying networks. To determine the grouping of individual connections we used hierarchical clustering with a weighted distance (across both the start and end points of the connections) algorithm and an average distance of 15 mm as the cutoff threshold in the clustering.

### Visualization of all-to-all connectivity

Significant coherence modulations at the level of the cortical grid were visualized on the cortical surface of an atlas brain using FreeSurfer. For visualization of the connections we used a circular plotting scheme [30] in which the nodes were based on an anatomical parcellation (AAL, Fig 2; [31]). For this purpose, we transformed the coordinates of the individual subjects to MNI (Montreal Neurological Institute) coordinates. MNI coordinates were obtained by combining the linear transformations between the head and Freesurfer MRI coordinates (6-parameter rigid body, obtained from the Elekta Neuromag software) and between the FreeSurfer MRI of an atlas brain (MNI306) to MNI coordinates (12-parameter affine, obtained from FreeSurfer) [32]. The data were represented by a reduced adjacency matrix *C*, which was constructed by assigning an anatomical label to the connection start and end point, such that the value of matrix element *C(i*,*j)* corresponded to the number of significant connections between anatomical areas *i* and *j*.

To quantify the lateralization of the networks we calculated a lateralization index LI for the number of connections within each hemisphere separately for words and symbols: LI = (left—right) / (left + right). The cut points LI>0.2 and <0.2 (i.e. at least 50% more results were found in one hemisphere than in the other) were considered to indicate left and right laterality, in concordance with previous studies [33].

### Network hubs and frequency-specific connections

Brain regions that interact with many other regions (hubs) are considered to be important for the integration of information within functional networks [34]. We used a common graph-theoretical measure of centrality, degree, to assess the importance of individual nodes. The degree was calculated as the total number of connections to a particular node [35] using unweighted adjacency matrices. We defined network hubs as nodes with a degree greater than the network mean plus one standard deviation [35]. The relative importance of each frequency band was determined by calculating the frequency-resolved degree across all brain regions: the number of connections were summed across all regions at each frequency band and divided by the total number of connections across all frequencies.

### Statistical evaluation of all-to-all connectivity

**Fig 3:**
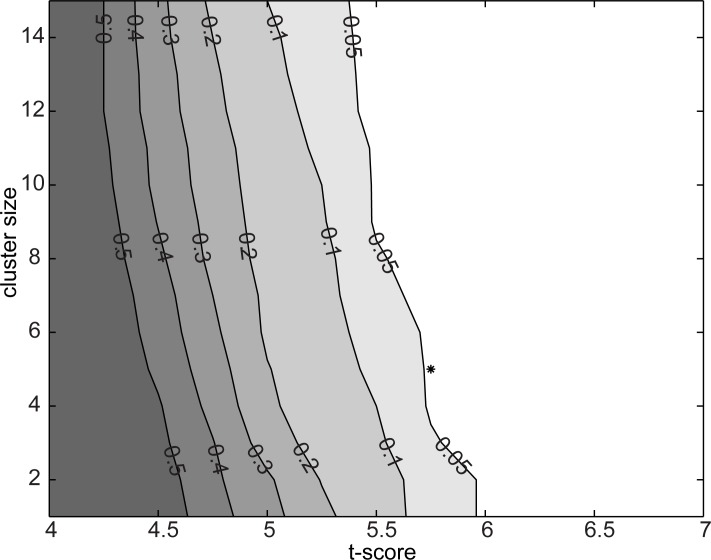
Estimation of appropriate statistical threshold. Each contour line depicts the likelihood level that a random data set, in this experimental setup, would yield a chance finding. The selected parameter set (t-score = 5.75, cluster size = 5), with a probability of chance findings of 0.05 or less, is indicated by a star.

Statistical evaluation of the connectivity results followed a procedure designed to address the possibility of chance findings specific to the spatial profile of the evaluated connectivity, MEG sensor configuration and subjects within this study (same type of MEG sensors at similar positions with respect to the subjects’ heads) [3]. The evaluation was based on simulated data and it addressed simultaneously the possibility of chance findings due to randomly occurring differences in true inter-regional coupling as well as due to randomly occurring systematic differences in field spread [28]. The parameters of the simulations were set to match the experimental data (the same amount of trials in both) and to elicit chance findings at a probability that would be at least equal to that in the experimental data (e.g., the simulated data consisted of a high number of simultaneously active sources that could, by chance, show systematic differencs between conditions). First, we generated 200 sets of simulated data for each subject consisting of 20 randomly placed cortical sources active at a random frequency between 0 and 75 Hz, with the number of trials matched to the real data. To determine the grouping of individual connections we used hierarchical clustering with a weighted distance (across both the start and end points of the connections) algorithm and an average distance of 15 mm as the cutoff threshold in the clustering. These were the same parameters as used in the analysis of the experimental data. Next, we calculated the statistical significance (paired samples t-tests) of change in coherence across two conditions for each connection. For t-scores ranging from 4 to 7 (0.25 step-size) and with cluster sizes ranging from 1 to 15 (1-connection step-size), we then evaluated whether the simulation would have produced any significant connections. This approach is similar to maximum-statistics permutation testing [36, 37]. It would also be possible to use other statistics than t-tests as long as the same statistics is used both to determine the significance threshold and to evaluate the real experimental data. From the 200 simulations we obtained a distribution of findings at each threshold pair (significance and cluster-size) and calculated the likelihood that a random data-set would produce any finding (Fig 3). For the analysis of the experimental data, we selected a parameter set at p = 0.045 approximately in the middle of this range (t-score = 5.75, cluster size = 5) and used it in all the subsequent analyses of the experimental data. The present approach for evaluating the significance of the all-to-all connectivity was chosen as it facilitates FWE type correction for MEG source-level estimates where a true neural connection can manifest as multiple linked connection estimates.

## Results

### Stimulus-specific modulations of coherence and network hubs

Statistically significant (p < 0.05, FWE) stimulus-specific changes in the all-to-all connectivity are presented for the five accepted frequency bands of interest: 3–7 Hz (theta), 8–13 Hz (alpha), 21–29 Hz (high beta), 30–45 Hz (low gamma), and 60–90 Hz (high gamma). The low beta band was excluded from the analysis due to power differences between conditions (cf. Methods).

**Fig 4:**
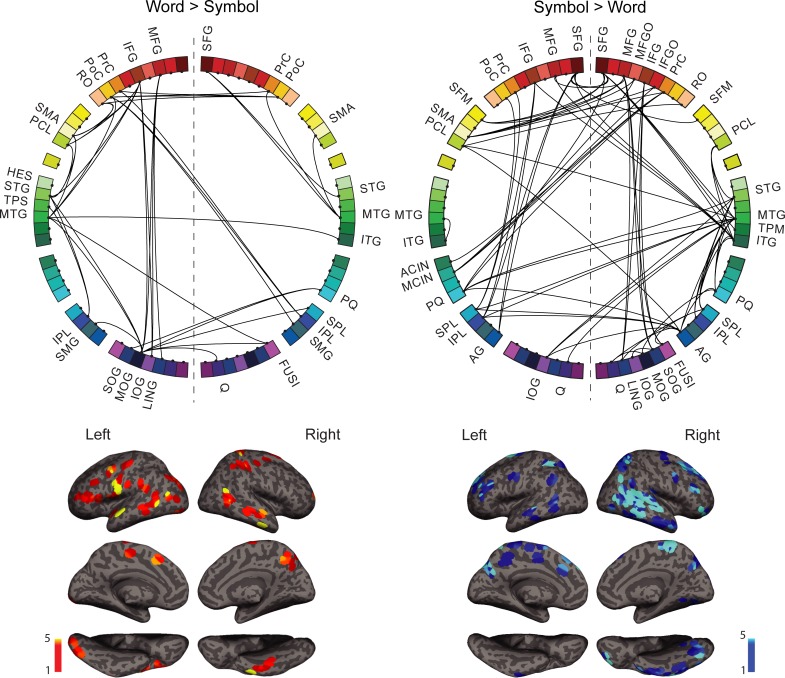
Spatial distribution of the stimulus-specific networks. Above: The overall stimulus-specific network modulations across all frequency bands for Words>Symbols (left) and Symbols>Words (right) shown on the circular representation; for cortical parcellation and region indices, see Fig 2. Below: The corresponding anatomical locations of the connection start and end points on the cortex for Words (left panel) and Symbols (right panel). All frequencies are overlaid.

**Fig 5:**
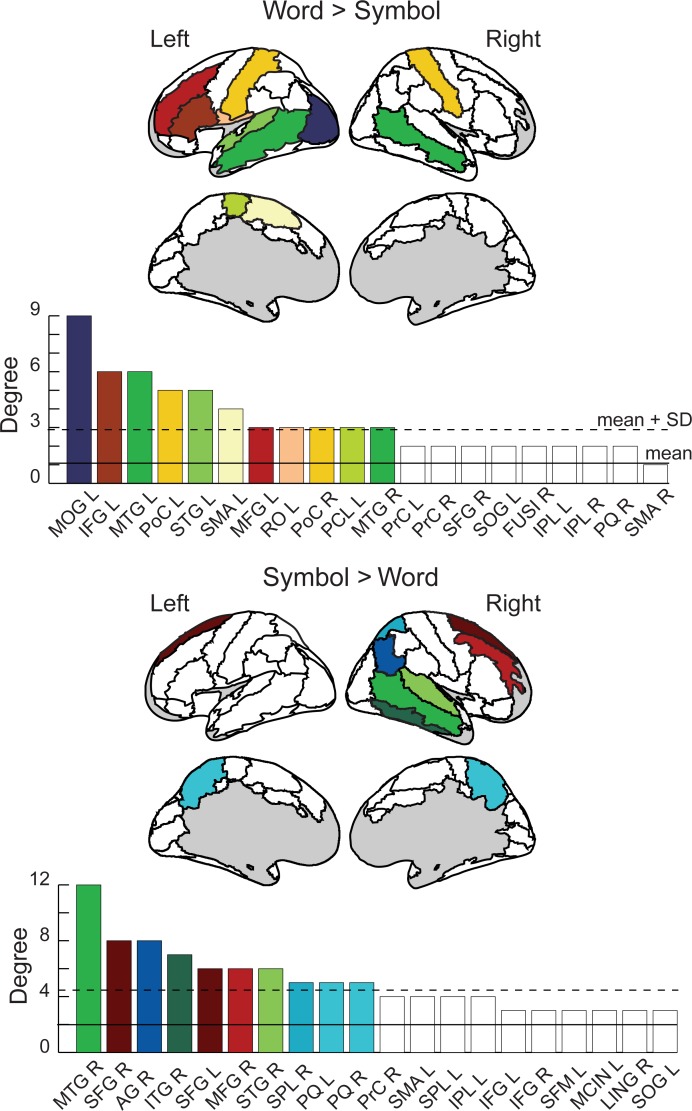
Network hub regions. High-degree nodes (shown in color on the AAL brain image; see Fig 2) are defined as exceeding mean (filled line) + 1 SD (dashed line). Degree is calculated across all frequencies. Network nodes are labeled according to the AAL parcellation scheme (Fig 2), and according to hemisphere (e.g. SMA R = supplementary motor area, right; SMA L = supplementary motor area, left). The regions are listed in order of descending degree. The 20 regions with the largest degree values are shown.

For written words (Fig 4, left), enhanced coherence with respect to symbol strings was detected in a left-hemisphere dominated network (lateralization index, LI = 0.2). Network hubs for words (Fig 5, top) were identified in the left middle occipital gyrus (MOG), the left superior and bilateral middle temporal gyrus (STG, MTG), the left inferior and middle frontal cortex and rolandic operculum (IFG, MFG, RO), bilateral post-central gyri (PoC), and medially in the left supplementary motor area and the paracentral lobule (SMA, PCL). The language regions that were of particular interest here, the left occipito-temporal region (anterior part of the MOG region), the left superior/middle temporal cortex (STG, MTG), and the left inferior frontal cortex (IFG) were all interconnected (Fig 4, left). The network also included regions within the motor and somatosensory cortex. The regions around the left central sulcus (pre- and post-central sulcus, rolandic operculum, PrC, PoC, RO), including the face motor and sensory regions, were connected with the corresponding right-hemisphere regions around the central sulcus (PrC, PoC) and with the left SMA.

For symbols (Fig 4, right), coherence was enhanced in a right-lateralized network (LI = -0.38). Network hubs for symbols (Fig 5, bottom) included right temporal regions (superior, middle and inferior temporal gyri (STG, MTG, ITG), frontal regions (bilateral superior frontal gyri, right middle frontal gyrus (SFG, MFG)), right parietal regions (angular gyrus and superior parietal lobule (AG, SPL), and bilateral precuneus (PQ).

### Frequency-specificity of the network modulations

**Fig 6:**
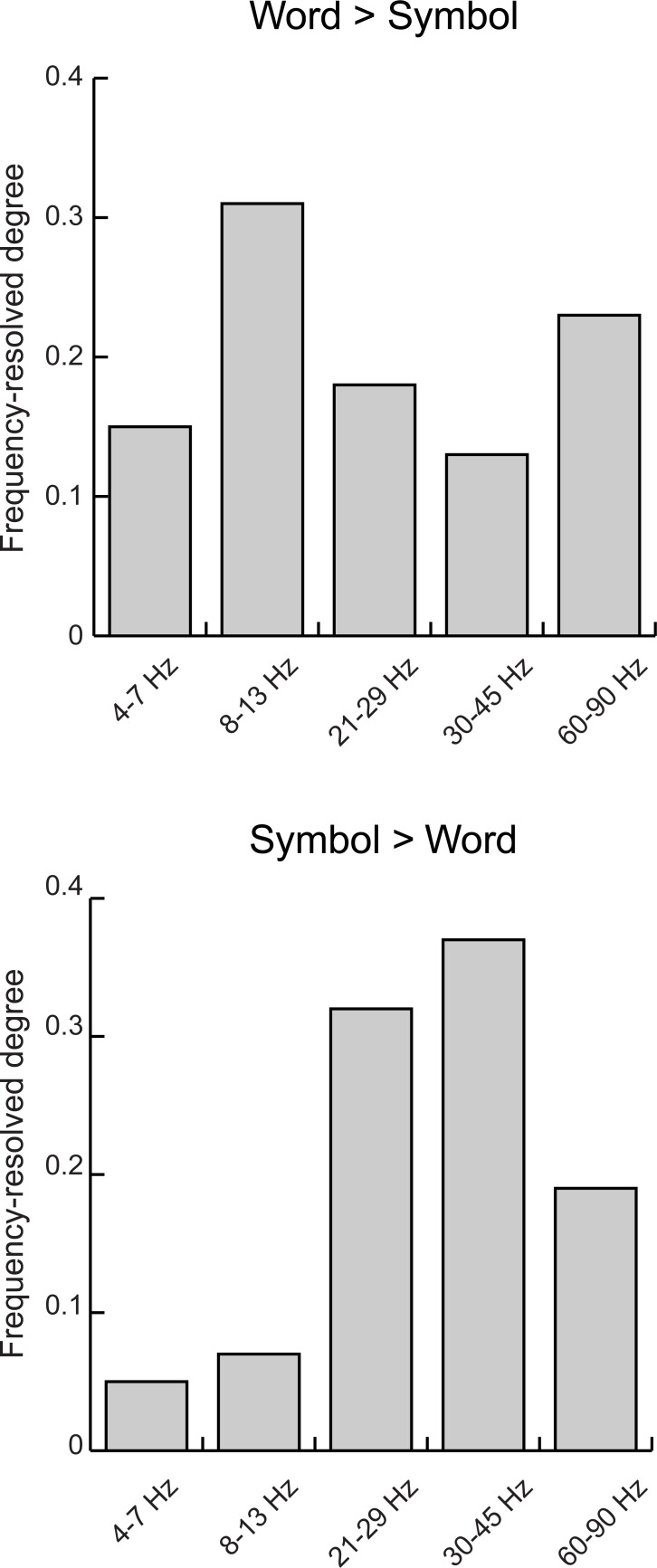
Frequencey-resolved degree. The relative importance of each frequency band for Words>Symbols and Symbols>Words, across all brain regions.

The spectral components showing the largest number of connections with enhanced coherence (frequency-resolved degree) were different for the word and symbol string stimuli (Fig 6). For the written words, increased coherence was detected in particular in the 8–13 Hz alpha band (31% of all significant connections) and in the 60–90 Hz high gamma band (23% of all significant connections). For symbol strings, coherence increases were detected particularly in the 21–29 Hz (high beta, 32% of the significant connections) and 30–45 Hz band (low gamma, 37% of the significant connections).

**Fig 7:**
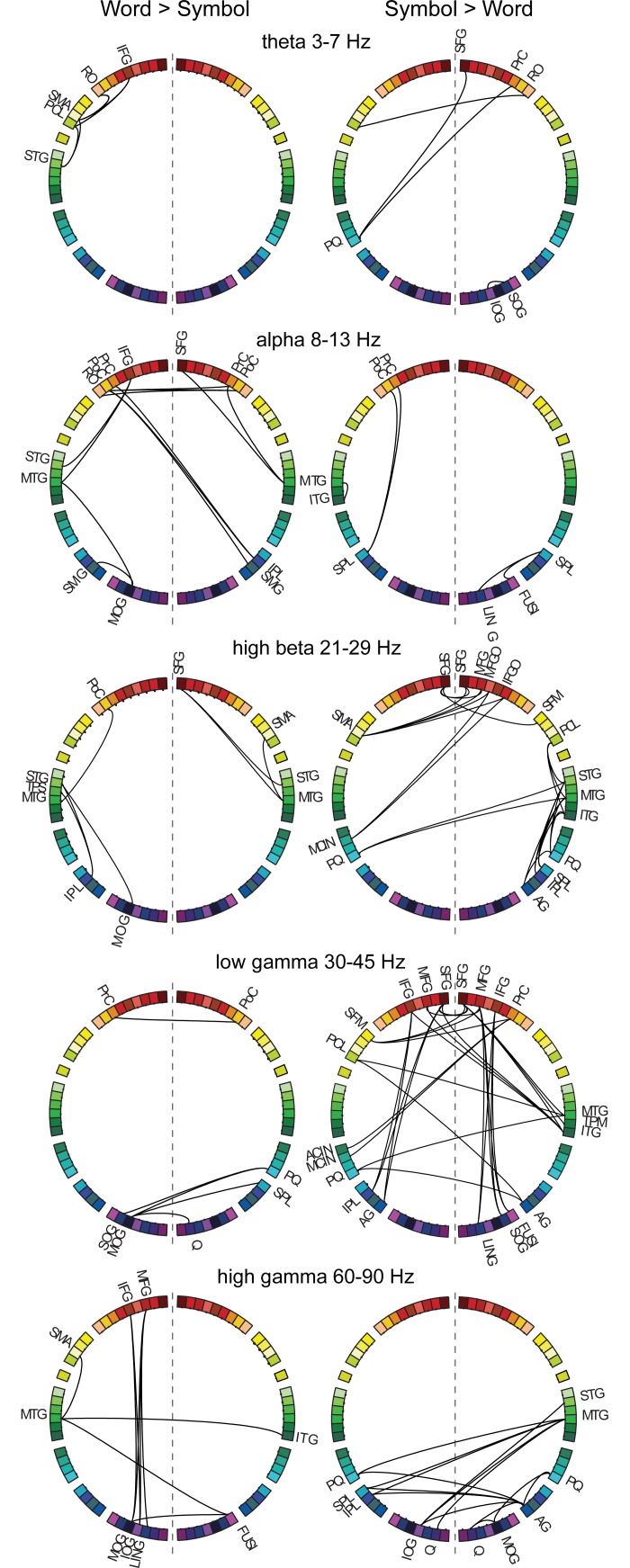
Frequency-resolved networks. Stimulus-specific networks at different frequency bands: 3–7 Hz (theta), 8–13 Hz (alpha), 21–29 Hz (high beta), 30–45 Hz (low gamma), and 60–90 Hz (high gamma) for Words>Symbols and Symbols>Words. For abbreviations, see Fig 2.

For words compared to symbols (Fig 7, left), the 8–13 Hz (alpha) band revealed connections between the classical language regions in the left hemisphere: the inferior frontal gyrus (IFG) was connected to the left anterior and posterior temporal cortex (STG, MTG). The left middle temporal and supramarginal gyri (MTG, SMG) were also connected to the left occipital cortex (MOG). Interhemispheric connections were detected between left-hemisphere regions around the central sulcus (left RO, PrC, PoC) and right parieto-frontal regions (PrC, PoC, IPL, SMG). In the right hemisphere, the middle temporal gyrus (MTG) was connected with the pre-central gyrus (PrC) and superior frontal gyrus (SFG). Within the 21–29 Hz (high beta) frequency band, the left superior temporal cortex (STG, TPS) was interconnected with the left middle occipital cortex and parietal cortex (MOG, IPL). The middle temporal cortex (MTG) was connected to the post-central gyrus in the left hemisphere, whereas in the right hemisphere connections were observed between the temporal cortex (STG, MTG) and the superior frontal and supplementary motor area (SFG, SMA). In the 60–90 Hz (high gamma) band, connections were detected between the left frontal regions (MFG, IFG) and the left occipital regions (MOG, SOG, LING). The left middle temporal gyrus (MTG) was connected with the left supplementary motor area (SMA) and the right inferior temporal/fusiform gyrus (ITG, FUSI) which was further connected to the left occipito-temporal cortex (MOG). The two remaining frequency bands highlighted links (3–7 Hz theta band) between the left medial cortex (SMA, PCL) and the left inferior frontal cortex (IFG, RO) and superior temporal cortex (STG), as well as (30–45 Hz low gamma) inter-hemispheric connections that linked, on the one hand, the left pre-central gyrus (PrC) and the right postcentral gyrus (PoC) and, on the other hand, the left occipital regions (MOG, IOG) and the right hemisphere parieto-occipital regions (cuneus, precuneus, superior parietal lobule, C, PQ, SPL).

For symbols compared to words (Fig 7, right), frontal regions (bilateral SFG, MFG, IFG) were inter-connected and also connected with the right temporal regions (MTG, ITG) in the 30–45 Hz band. In addition, within hemisphere, left frontal regions (SFG, IFG, PrC, PoC) were connected with the left parietal regions (IPL, AG), and right frontal regions (MFG, IFG) were connected with the right occipito-temporal regions (lingual gyrus, inferior occipital gyrus, fusiform gyrus (LING, IOG, FUSI)). Interhemispheric connections were detected between the right temporo-parietal regions (PoC, MTG, AG) and left medial structures (medial frontal gyrus, anterior and middle cingulate gyrus, precuneus, paracentral lobule (FM, CIA, CINM, PQ, PCL)). In the 21–29 Hz (high beta) band, right temporal regions (STG, MTG, ITG) were connected with the right parietal cortex (SPL, IPL, AG), the bilateral precuneus (PQ) and the right paracentral lobule (PCL). Connectivity between the frontal cortex (bilateral SFG, right MFG, MFGO, IFGO) and the medial cortex (right SFM, left SMA, MCIN, PQ) was also detected. In the 60–90 Hz (high gamma) band, the left parietal regions (SPL, IPL, PQ) and left occipital regions (cuneus, superior occipital gyrus, (Q, SOG)) were connected with the right parieto-temporal regions (STG, MTG, AG). The right angular gyrus and middle occipital gyrus also had connections with the right precuneus and cuneus. The relatively few significant connections found at low frequencies indicated links (8–13 Hz alpha) between the left superior parietal gyrus (SPL) and left pre- and postcentral gyrus (PrC, PoC), right superior parietal gyrus (SPL) and right lingual and fusiform gyri (LING, FUSI), left middle and inferior temporal gyri (MTG, ITG), as well as links (3–7 Hz theta) between right-hemisphere frontal regions (SFG, PrC, RO) and the left precuneus and paracentral lobule (PQ, PCL).

## Discussion

To discover the reconfiguration of large-scale brain networks during visual recognition of different form categories, we determined MEG-derived functional connectivity for word reading and processing of symbol strings. Large-scale functional networks are often identified from fMRI resting state measurements [38], and more recently also from MEG resting state measurements [39]. However, the typical reading network is generally not prominent in a data-driven all-to-all connectivity analysis of resting state data [40, 41], although it may be detected with seed-based focus on specific language-related regions [42]. One reason for the low visibility of the reading network might be that reading is a relatively recent skill that does not rely on a built-in network of regions that would emerge in a resting state analysis [17, 43]. Thus, identification of the reading network might be markedly enhanced by using experimental manipulation to drive the system into a state predominated by stimulus-specific interactions [30]. In the present study, all-to-all connectivity mapping of cortical coherence revealed spatially distinct connectivity patterns for words and symbols that relied on oscillatory communication in different frequency bands. The present study thus demonstrates the formation of frequency-resolved large-scale network patterns driven by input stimuli and provides novel evidence that such networks are flexibly modulated to support specific behaviors.

### Coherence as a mechanism for neural interactions

We used frequency-specific oscillatory signals as measured by MEG as markers of the underlying network interactions. Synchronized oscillatory activity of distant neural populations has been suggested to form large-scale, temporary connections in the brain [1]. Within this framework, transient functional connectivity via synchronized oscillations enables integration of information across spatially distributed regions, flexibly changing which regions participate in the network [2, 3, 44]. Here, frequency-specific networks were observed, with the largest coherence increase for words vs. symbols in the 8–13 Hz and 60–90 Hz bands, and for symbols in the 21–29 Hz and 30–45 Hz bands. It has been suggested that the same region can participate in communication at several frequencies simultaneously [44, 45]. In agreement with this, in the present data, many of the same nodes showed synchronization modulations at both gamma and lower frequency bands, e.g. within the left inferior frontal gyrus at 60–90 Hz and 8–13 Hz. Overall, the present results support the role of coherence in specific frequency bands as the mechanism for integrating task-relevant information across brain regions to facilitate both reading and visual recognition.

### Distinct signature networks for words and symbols

Category-specific information is thought to be processed within distinct regions in the occipito-temporal cortex [13–15]. Such regional specialization is thought to arise either through inherent structural connections of such regions, or through their preference for visual shapes [17]. For reading, it has been shown that a region that is important for visual recognition of words develops when reading skills are acquired [18]. Activation and anatomical connectivity studies suggest that, once formed, such regions could be important nodes in the large-scale networks that process visual input [17, 46, 47]. The present findings, obtained via evaluation of global large-scale functional connectivity patterns, support this notion. The results demonstrate that processing of different form categories, written words vs. symbol strings, leads to the formation of large-scale functional networks that operate at distinct oscillatory frequencies and that incorporate these regions, suggesting that category-specific processing should be viewed not so much as a local process but as a distributed neural process implemented in signature networks.

Overall, the findings align with the present view of parallel but interacting processing pathways underlying visual processing of objects [46, 48]. The identified network for symbols encompassed connections between occipital and parietal regions that are part of the dorsal stream of visual processing [48, 49], and between occipital and temporal cortex that are part of the ventral pathway of visual object recognition [46, 49], but also highlighted the role of the right middle temporal cortex as a hub in the network for processing symbols. For written words, enhanced coherence was detected in a left-hemisphere dominated network, with nodes in classical language regions: left inferior frontal, middle/superior temporal, supramarginal and occipito-temporal cortex. Previous studies have shown activation sequences proceeding from the occipital visual regions via the left occipito-temporal cortex towards anterior left-hemisphere language regions [5, 6, 50, 51]. Activations within these regions have been suggested to correspond to the involvement of two reading routes: a lexical reading route for stored lexical semantic information, and a non-lexical reading route for grapheme-phoneme conversion [52, 53]. Here, following the suggested grapho-phonological route, the left occipito-temporal cortex was found to be connected with the left parieto-temporal junction (supramarginal gyrus). Ventrally, following the suggested lexico-semantic route, the left occipital cortex was connected with the anterior temporal cortex that was further connected with the left inferior frontal gyrus. We also detected a connection between the left occipito-temporal cortex and the left inferior frontal gyrus, in line with previous ROI-based analysis suggesting existence of such a direct connection [10, 54–56]. Anatomically, these regions are connected via the arcuate fasciculus dorsally, and via the inferior fronto-occipital fasciculus and inferior longitudinal fasciculus ventrally that connect the occipital cortex to the left inferior frontal gyrus [57–59].

### Large-scale networks reflect the neural implementation of stimulus-task interplay

The network recruited when processing symbol strings was more right-lateralized than for real words, with prominent fronto-parietal connections. Activation within these fronto-parietal regions has been associated with direction of spatial attention and with visual search for task-relevant objects, usually with right-hemisphere dominance [60, 61]. In visual working memory, sustained synchronization in a large-scale cortical network involving frontal, parietal temporal and occipital brain regions has been observed during the retention period and shown to increase with memory load [62]. We asked our participants to indicate when the same stimulus occurred twice in a row (a one-back task). Our results, demonstrating synchronization between frontal and parietal regions mainly in the low gamma band may reflect the enhanced memory load in the symbol condition, thus supporting the role of synchronization as a mechanism for maintenance of object representation in visual working memory [62]. The present analysis also demonstrated increased involvement of a sensorimotor network in silent word reading, including regions within the left pre- and postcentral gyrus, the left SMA and right supramarginal/inferior parietal cortex. While this network has been primarily associated with motor [22] and speech production [3] tasks, our results, together with earlier connectivity results in reading [9], point to the involvement of the motor system in silent reading. This could reflect a partly different strategy evoked by word versus symbol stimuli to accomplish the same (one-back) task. Involvement of the motor network might thus relate to internal rehearsal of the presented word [63] in order to facilitate task performance.

Importantly, these findings suggest that performing a one-back task on different stimulus categories can be neurally implemented in separate networks, and thus bear important implications for future studies. Given the nature of the task (one-back task) and the stimuli (words vs. symbol strings), it is likely that the word condition relied more heavily on higher-level (linguistic) processing of the stimuli, whereas the symbol condition relied more heavily on low-level feature analysis and visual working memory. The present results may be interpreted as a differentiation between a ‘verbal working memory’ network for the word condition (involving language pathways and sensorimotor regions), and a ‘visual working memory’ network for the symbol condition (involving visual pathways and fronto-parietal connections), in analogy to the original Baddeley and Hitch model that proposed a division into a phonological loop for verbal working memory and a visuo-spatial sketchpad for visual working memory [64]. Similarly, engaging in a verbal vs. visuospatial strategy to perform the same working memory task has been shown to modulate task performance and brain activity [65], supporting the notion that different brain networks mediate different working memory strategies. Our results further suggest that coherence across brain regions is an important mechanism in the formation of such networks.
